# Supplementary figures and images for: Viral Epidemics in a Cell Culture: Novel High Resolution Data and Their Interpretation by a Percolation Theory Based Model
Source: PLoS One. 2010 Dec 20;5(12):e15571. doi: 10.1371/journal.pone.0015571 (PMC3004943; doi:10.1371/journal.pone.0015571)

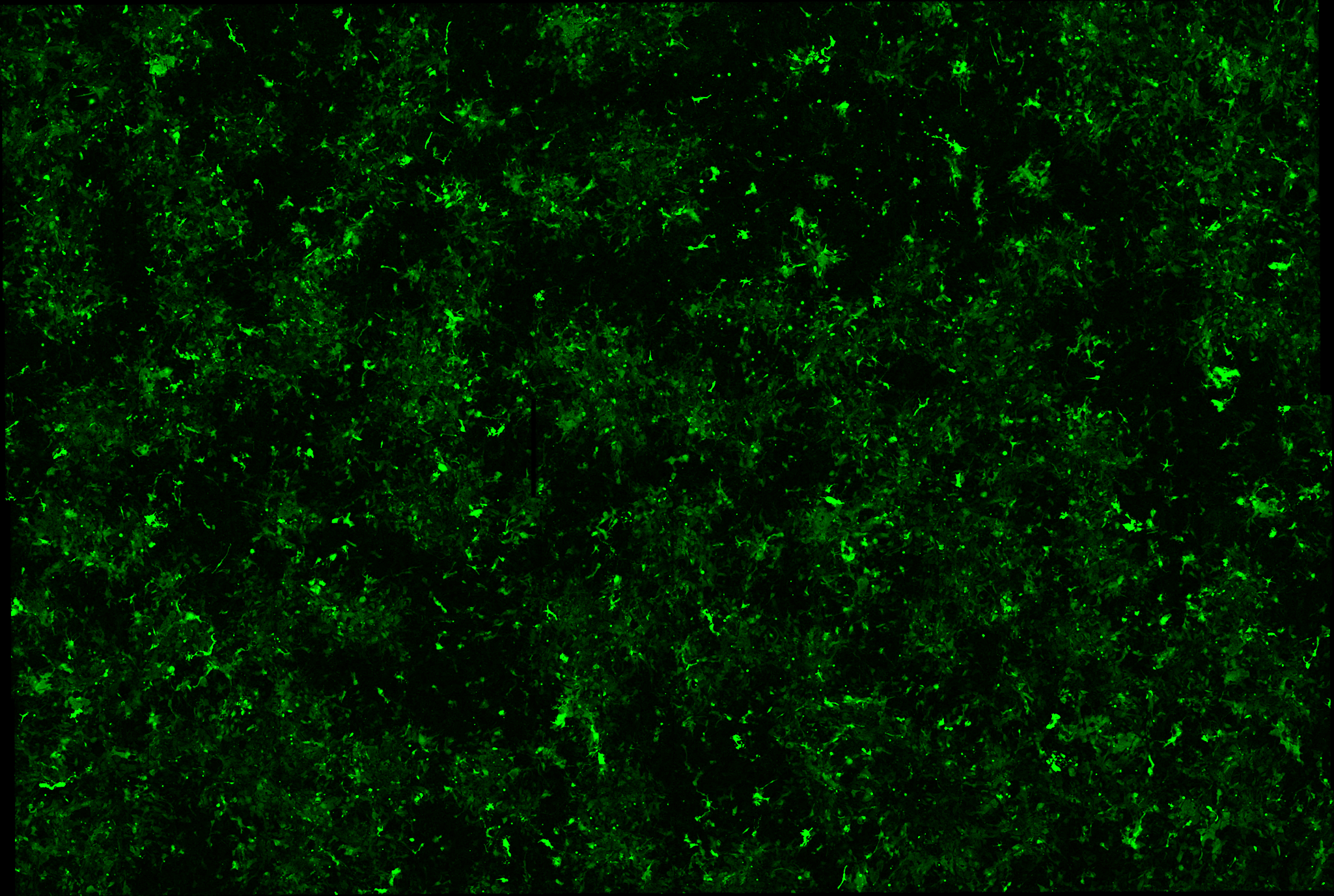

Supplement: Figure S1 — Original image of Figure 3 . 10×10 microscopic field images were taken from fixed cultures with GFP expressing infected cells, 48 hours after the onset of infection with “high” virus titer. High resolution file can be downloaded from: http://amur.elte.hu/BDGVirus/ (TIF) [file pone.0015571.s001.tif]

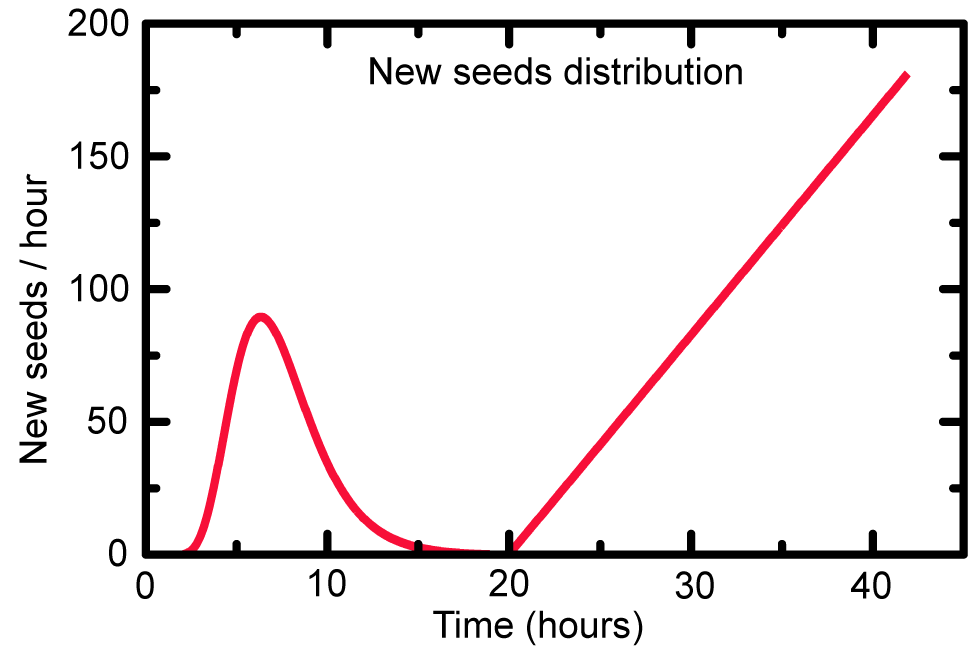

Supplement: Figure S2 — Rate of appearance of new cluster seeds as a function of time in the model. Simulation starts with 500 seeds with a “switching on” time following a log-normal distribution fitted to the experimentally observed parameters (mean =7.5 h, standard deviation =2.5 h), and continues with 2000 further seeds with a constantly increasing rate in time between 20 h and 42 h corresponding to the observed second wave of infection. (TIF) [file pone.0015571.s002.tif]

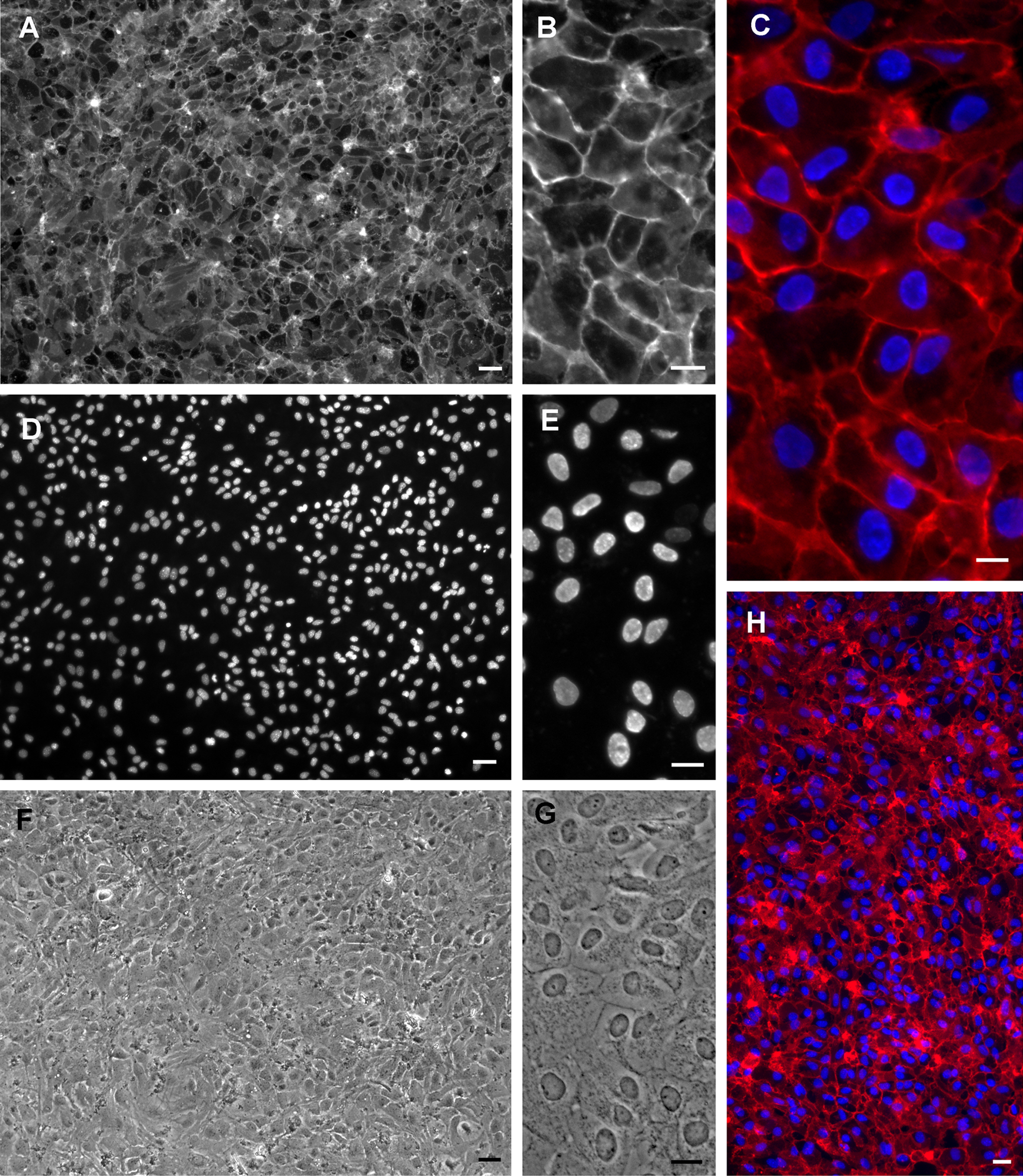

Supplement: Figure S3 — Features of the astrocytic cultures. Astrocytic cultures were live-stained with a fluorescent plasma membrane stain (A,B,C,H) and the nuclei were labeled with DAPI (C,D,E,H). Both the membrane-stained and the phase contrast (F,G) images show that astrocytes are arranged to a mosaic like monolayer. Scale bars: 25 µm (A,D,F,H), 10 µm (B,C,E,G). High resolution file can be downloaded from: http://amur.elte.hu/BDGVirus/ (TIF) [file pone.0015571.s003.tif]

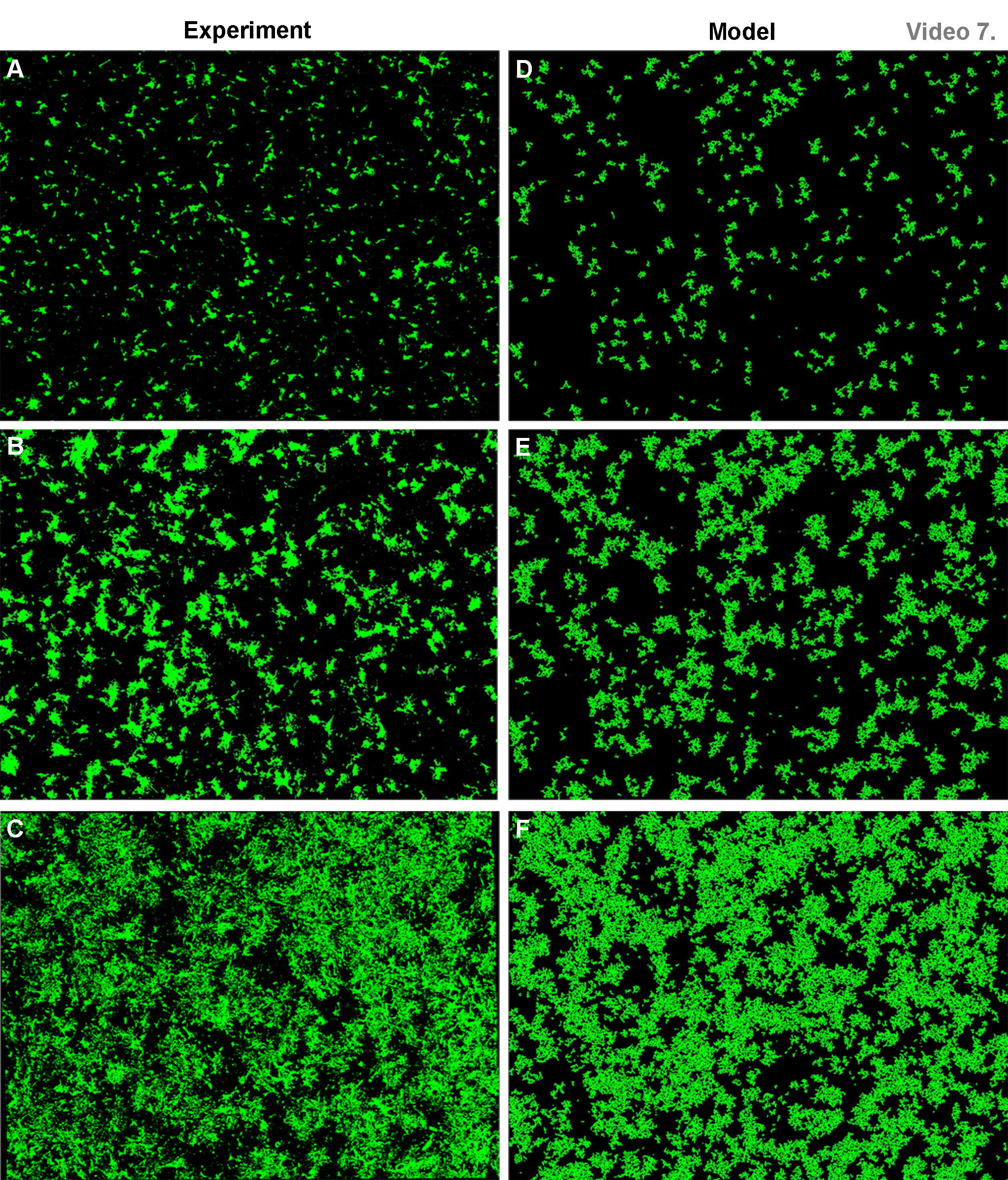

Supplement: Figure S4 — Comparing the experimentally and computationally obtained clusters. Comparison of clusters found by the Hoshen – Koppelman algorithm on photo montages (A,B,C) and clusters developed in the model (D,E,F). Time elapsed after infection was 18 h (A,D), 30 h (B,E) and 42 h (C,F), respectively. Cluster growth, imitating virus propagation in the model can be seen in supplementary Video S7. High resolution file can be downloaded from: http://amur.elte.hu/BDGVirus/ (TIF) [file pone.0015571.s004.tif]

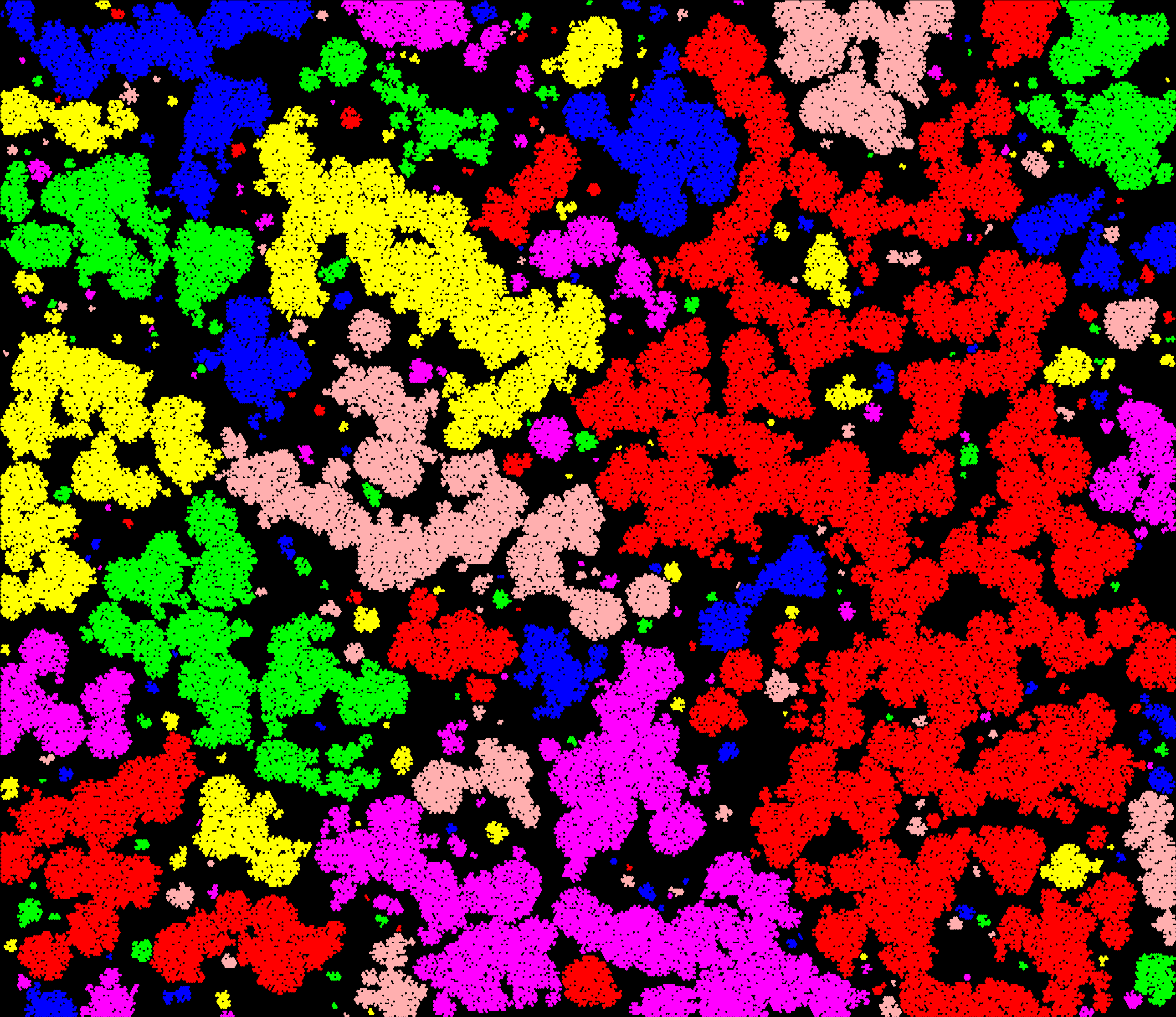

Supplement: Figure S5 — Clusters obtained in the simulations of a simplified model. The clusters generated with seeds of infection appearing at a rate corresponding to the experiment, but the infection spreading according to the p=0.7 simple percolation rule leads to images like this one. The clusters, on the scale being about 20 to 50 lattice units (a lattice unit corresponding to the size of a cell), are much more compact than the ones observed in the experiments, and their percolation-like features start to show up only at a much larger scale than in the experiments. (TIF) [file pone.0015571.s005.tif]
