# Supplementary material for: Viral Epidemics in a Cell Culture: Novel High Resolution Data and Their Interpretation by a Percolation Theory Based Model
Source: PLoS One. 2010 Dec 20;5(12):e15571. doi: 10.1371/journal.pone.0015571 (PMC3004943; doi:10.1371/journal.pone.0015571)
Supplement: Table S1 — Removal of infective viral particles from the culture medium. Percentage of infected (GFP expressing) cell groups developed in astrocytic cultures incubated with the first 3 washes of virus treated “original” sister‐cultures for 1 h or 24 hours. “Low titer”: 2,5×10^4^ PFU/ml, “High titer”: 2,5×10^5^ PFU/ml. (DOC) [file pone.0015571.s006.doc]

|  | Original  [%] | 1^st^ wash [%] | 2^nd^ wash [%] | 3^rd^ wash [%] |
| --- | --- | --- | --- | --- |
| Low titer, 1 h | 67 | 7 | 0 | 0 |
| Low titer, 24 h | 63 | 17 | 0 | 0 |
| High titer, 1 h | 100 | 10 | 7 | 0 |
| High titer, 24 h | 100 | 33 | 13 | 7 |
