# Supplementary material for: Viral Epidemics in a Cell Culture: Novel High Resolution Data and Their Interpretation by a Percolation Theory Based Model
Source: PLoS One. 2010 Dec 20;5(12):e15571. doi: 10.1371/journal.pone.0015571 (PMC3004943; doi:10.1371/journal.pone.0015571)
Supplement: Table S2 — Release of infective viral particles to the culture medium by astrocytes. Percentage of infected (GFP expressing) cell groups developed in astrocytic cultures incubated for 1 h or 24 hrs with conditioned media (CM) taken from virus treated sister‐cultures. nd: not detected (DOC) [file pone.0015571.s007.doc]

| High titer  CM | Original  [%] | 1 h  [%] | 24 h  [%] |
| --- | --- | --- | --- |
| 1 h | 0 | 0 | 0 |
| 5 h | 0 | 0 | 0 |
| 24 h | 63 | 0 | 7 |
| 30 h | 80 | 7 | 20 |
| 48 h | 100 | nd | 100 |
